# Sliding-mode control based on prescribed performance function and its application to a SEA-Based lower limb exoskeleton

**DOI:** 10.3389/frobt.2025.1534040

**Published:** 2025-03-04

**Authors:** Feilong Zhang, Tian Wang, Liang Zhang, Enming Shi, Chengchao Wang, Ning Li, Yu Lu, Bi Zhang

**Affiliations:** ^1^ State Key Laboratory of Robotics, Shenyang Institute of Automation, Chinese Academy of Sciences, Shenyang, China; ^2^ University of Chinese Academy of Sciences, Beijing, China; ^3^ The Fourth Affiliated Hospital of China Medical University, Shenyang, China; ^4^ Department of Rehabilitation Medicine, The People’s Hospital of Liaoning Province, Shenyang, China

**Keywords:** prescribed performance, sliding-mode control, predefined convergence zone, lower limb exoskeleton, transient characteristics

## Abstract

A sliding-mode control based on a prescribed performance function is proposed for discrete-time single-input single-output systems. The controller design aims to maintain the tracking error in a predefined convergence zone described by a performance function. However, due to the fixed structure of the controller, the applicability and universality of this method are limited. To address this issue, we separate the controller into two parts and analyze the principle of the prescribed performance control (PPC) method. Then we can replace the linear part of the controller with model-based control methods to adapt to the specific characteristics of the controlled system. Compared with current works, when the established system model is inaccurate, we can enhance the smoothness or response speed of the system by introducing a penalty constant to alter the system’s transient characteristics while the tracking error is within the prescribed domain. Finally, numerical comparison simulations and a lower limb exoskeleton experiment illustrate the established results and the effectiveness of the proposed method.

## 1 Introduction

Most control methods are performance-based methods. For example, the parameters of the PID are designed either to obtain the desired performance of rise time, maximum overshoot and steady-state error of the unit-step response of the system, or to achieve the desired sheared frequency and phase margin through Nichols plot or Bode diagram. If the performance of higher-order system cannot be guaranteed by PID controller, we can not only introduce the state/output feedback control and self-tuning method to achieve the desired locations of the poles which determine the speed and damping of the system response, but also apply the LQR, minimum variance control and predictive control methods to acquire the optimal control performance (Mir and Senroy, 2020; Kim and Ahn, 2018; Tajaddodianfar et al., 2019; Liang et al., 2019; Ahmed et al., 2017; Rosolia and Borrelli, 2017). Additionally, in consideration of some systems with the slight nonlinearity, parameter uncertainty and even unknown structures, the performance of adaptability is made possible through combining the online estimate algorithm with the above controller according to the certainty equivalence principle (Åström and Wittenmark, 2013; Goodwin and Sin, 2009; Zhou et al., 2018; Zheng et al., 2016; Xi et al., 2024; Chen et al., 2021).

Considerable efforts have been made to deal with the design of the prescribed performance controller (PPC) for a kind of output constrained control problem. This methodology originates from (Bechlioulis and Rovithakis, 2008) and means that the tracking error should converge to an arbitrarily small residual set. Its advantage is that both transient and steady-state performance of the system can be assured, i.e., the convergence rate is no less than a prespecified value and the maximum overshot is less than a small prespecified domain (Bechlioulis and Rovithakis, 2009; Bechlioulis and Rovithakis, 2011; Bechlioulis and Rovithakis, 2014). In addition, a series of state feedback control schemes are proposed in combination with fault tolerance (Gao et al., 2022), fuzzy adaptive (Chen et al., 2021; Sui et al., 2021), finite-time index (Dong and Yang, 2022; Gao et al., 2021; Liang et al., 2022), and neural network finite-time index (Shi et al., 2024; Deng et al., 2022) for realization of the PPC.

The aforementioned PPC methods are based on continuous state space model, however, sometimes discrete-time control methods are more difficult to analyze and are more suitable for computer control in industrial setting. A kind of sliding mode control based on ARX model was proposed by Nguyen et al. (2017) to maintain the tracking error trajectory in a predefined convergence zone described by the performance function. Similarly, (Liu and Yang, 2018; Liu and Yang, 2019a; Liu and Yang, 2019b), propose one class of sliding mode control based on the equivalent dynamic linearization model and introduce the online identification for the adaptability of this class of method in nonlinear systems (Zhang et al., 2020; Zhang et al., 2021). Generally speaking, we shall begin with the topic on the certain linear system with ARX model like Nguyen et al. (2017) for more easily understanding the principle of this class of discrete PPC method (Nguyen et al., 2017; Liu and Yang, 2018; Liu and Yang, 2019a; Liu and Yang, 2019b), because the adaptability for the uncertainty or nonlinearity is radically introduced by on-line parameters estimation according to certainty equivalence principle Åström and Wittenmark, 2013; Goodwin and Sin, 2009). In addition, Nguyen et al. (2017) mainly focuses on the tracking performance, which is determined by performance function, and disregards the transient characteristics which can be changed through the adjustment of the parameters in controller. Actually, the prescribed performance is not the only index of system performance, and many well-known and visible transient performance indexes of the system should be concerned.

This paper proposes a new sliding mode control with a prescribed performance function. To enhance the applicability and universality of the PPC method, we separate the proposed controller into two parts: the linear feedback part and the nonlinear part. The linear part stabilizes the system by placing the poles of the system at the origin point to hold the system output and satisfy the optimal control index. Meanwhile, the actual output zone is enlarged by adding the nonlinear part of the controller, while the output error is still guaranteed within the prescribed zone defined by Nguyen et al. (2017) and Liu and Yang (2018), Liu and Yang (2019a), Liu and Yang (2019b). More precisely, simulations show that the linear part is the prerequisite for guaranteeing the convergence of tracking error within a predefined arbitrarily small zone. At the same time, the maximum overshot is less than the desired constant.

The main contributions are summarized as follows: i) To address the issue where the linear part of the controller cannot ensure sufficiently small errors under conditions of inaccurate modeling, we modify the denominator of the controller by introducing a penalty constant to tune the system behaviors. In this way, we can guarantee the system’s output within the prescribed zone and tune the introduced penalty constant to further improve the other system’s transient characteristics, such as smoothness, response speed, overshoot, etc. ii) We replace the linear part of the PPC controller with a dynamic model of the exoskeleton, and propose an incremental PPC controller based on PID + dynamics to ensure that the tracking error remains within a predefined region. The proposed control method is then successfully applied on a lower limb exoskeleton.

The rest of the paper is organized as follows: Section 2 provides the problem formulation and preliminaries. Section 3 introduces the design procedure and stability analysis of the proposed method. Section 4 presents simulations to study the PPC method and an experiment to test the controller. The conclusion is given in Section 5.

## 2 Problem formulation and preliminaries

### 2.1 System description

Consider the single-input and single-output discrete-time linear dynamical system (Equation 1) as follow:

$$A\left(z^{-1}\right)y\left(k\right)=B\left(z^{-1}\right)u\left(k\right)+\zeta \left(k\right)$$ (1)

where $u\left(k\right)$ is the control input; $y\left(k\right)$ is the measured output; $\zeta \left(k\right)$ is the unknown modeling errors and nonlinearities. $A\left(z^{-1}\right)$ and $B\left(z^{-1}\right)$ are defined as Equation 2

$$\begin{array}{cc} A\left(z^{-1}\right) & \;=1+a_{1}z^{-1}+a_{2}z^{-2}+\cdots +a_{na}z^{-na}\;\\ B\left(z^{-1}\right) & =b_{1}z^{-1}+\cdots +b_{nb}z^{-nb} \end{array}$$ (2)

where *n*_*a*_ and *n*_*b*_ are the orders of system output and control input, respectively.

Assumption 1 *The unmodeled dynamics* $\zeta \left(k\right)$ is slowly varying with respect to the sampling frequency and the unmodeled dynamics estimation error satisfies the condition that $\left|\tilde{\zeta }\left(k\right)\right|\leq \delta$ , where δ is a small positive number.

### 2.2 Prescribed performance function

The prescribed performance control method proposed by Nguyen et al. (2017) and Liu and Yang (2018), Liu and Yang (2019a), Liu and Yang (2019b) is designed to ensure adherence to specified tracking error constraints (Equation 3) as follows:

$$-\rho_{k}{\underline{\chi }}_{k}<e\left(k\right)=y_{d}\left(k\right)-y\left(k\right)<\rho_{k}{\bar{\chi }}_{k}$$ (3)

where *y*_*d*_(*k*) represents the desired output of the system at the time *k*; ${\underline{\chi }}_{k}$ and ${\bar{\chi }}_{k}$ represent the lower and upper bounds, respectively. $\rho_{k}$ is a bounded and strictly positive decreasing function with the property (Equation 4) as follow:

$$\lim_{k\to \infty }\rho_{k}=\rho_{\infty }$$ (4)

and have given the performance function and the upper and lower bounds (Equations 5–7) as follow:

$$\rho_{k+1}=\kappa \rho_{\infty }+\left(1-\kappa \right)\rho_{k}$$ (5)

$${\bar{\chi }}_{k+1}=\kappa +\left(1-\kappa \right){\bar{\chi }}_{k}$$ (6)

$${\underline{\chi }}_{k+1}=\kappa +\left(1-\kappa \right){\underline{\chi }}_{k}$$ (7)

where $\rho_{0}\geq \rho_{k}\geq \rho_{\infty }\geq 0$ and 0<κ<1 .

To address the constrained control issue (Equation 3), we transform the tracking error $e\left(k\right)$ into an unconstrained equivalent form and give a strictly increasing function $\Theta \left(\tau_{k}\right)$ of a transformed error $\tau_{k}$ . Define

$$e\left(k\right)=\rho_{k}\Theta \left(\tau_{k}\right)$$ (8)

The strictly increasing function must satisfy the following conditions (Equations 9, 10).

$$\Theta \left(\tau_{k}\right)\in \left(-{\underline{\chi }}_{k},{\bar{\chi }}_{k}\right)$$ (9)

$$\lim_{\tau_{k}\to +\infty }\Theta \left(\tau_{k}\right)={\bar{\chi }}_{k}\text{ and }\lim_{\tau_{k}\to -\infty }\Theta \left(\tau_{k}\right)=-{\underline{\chi }}_{k}$$ (10)

Due to the above properties of $\Theta \left(\tau_{k}\right)$ and $\rho_{k}\geq \rho_{\infty }>0$ , the inverse transformation can be obtained by Equation 11

$$\tau_{k}=\Theta^{-1}\left(\frac{e\left(k\right)}{\rho_{k}}\right)$$ (11)

With regard to the given tracking error $e_{0}$ , if $\rho_{0}$ is selected such that $-{\underline{\chi }}_{0}\rho_{0}<e\left(0\right)<\rho_{0}{\bar{\chi }}_{0}$ and $\tau_{k}$ is bounded, then $\Theta \left(\tau_{k}\right)\in \left(-{\underline{\chi }}_{k},{\bar{\chi }}_{k}\right)$ holds and (Equation 3) is guaranteed.

We introduce a strictly increasing function (Equation 12) for control design.

$$\Theta \left(\tau_{k}\right)=\frac{{\bar{\chi }}_{k}e^{b\tau_{k}}-{\underline{\chi }}_{k}a}{e^{b\tau_{k}}+a}$$ (12)

Then we have the transformed error $\tau_{k}$ as follows:

$$\tau_{k}=\frac{1}{b}\ln a\left(\frac{{\underline{\chi }}_{k}\rho_{k}+e\left(k\right)}{{\bar{\chi }}_{k}\rho_{k}-e\left(k\right)}\right)$$ (13)

When *a* = 1 and *b* = 2, (Equation 13) will degenerate into the transformed error in Nguyen et al. (2017).

The above prescribed performance function design in Liu and Yang (2018), Liu and Yang (2019a), Liu and Yang (2019b) is designed for the system without consideration of unmodeled dynamics, then Nguyen et al. (2017) further modified the tracking error constraint (Equation 3) into

$$-\delta -\rho_{k}{\underline{\chi }}_{k}<e\left(k\right)<\rho_{k}{\bar{\chi }}_{k}+\delta$$ (14)

Which takes the unmodeled dynamics including the disturbance and nonlinearities and offset error into consideration. *δ* is a small constant (Nguyen et al., 2017).

## 3 Prescribed performance control design

Consider the following sliding mode function (Equation 15):

$$s\left(k\right)=\theta \left(k\right)-1$$ (15)

where $\theta \left(k\right)$ is a variable derived from the transformed error and is defined as Equation 16

$$\theta \left(k\right)=\frac{{\underline{\chi }}_{k}\rho_{k}+e\left(k\right)}{{\bar{\chi }}_{k}\rho_{k}-e\left(k\right)}$$ (16)

Then the reaching condition is given as follows:

$$s\left(k+1\right)-s\left(k\right)=-qTs\left(k\right)-\Lambda_{s}sign\left(s\left(k\right)\right)$$ (17)

where $\Lambda_{s}>0$ . In this note, we adopt

$$\Lambda_{s}=qT$$ (18)

We can obtain the one step-ahead tracking error as follows

$$\;e\left(k+1\right)=y_{d}\left(k+1\right)-y\left(k+1\right)$$

$$=y_{d}\left(k+1\right)+a_{1}\left(k\right)+\cdots a_{na}y\left(k-n_{a}+1\right)$$ (19)

$$\;-b_{1}u\left(k\right)-\cdots b_{nb}u\left(k-n_{b}+1\right)-\zeta \left(k-1\right)$$

Here, we define $\hat{\zeta }\left(k\right)$ as the estimated unmodeled dynamics. According to the discrete-time perturbation estimation technique (Nguyen et al., 2017), we can estimate this unknown term by one-step delayed value as follows:

$$\begin{array}{ll} \zeta \left(k+1\right) & \approx \zeta \left(k\right)=y\left(k\right)+a_{1}y\left(k-1\right)+\cdots +a_{na}y\left(k-n_{a}\right)-b_{1}u\left(k-1\right)\\ & \;-\cdots b_{nb}u\left(k-n_{b}\right) \end{array}$$ (20)

We define the perturbation estimation error $\tilde{\zeta }\left(k\right)$ as follow

$$\tilde{\zeta }\left(k\right)=\hat{\zeta }\left(k\right)-\zeta \left(k\right)$$ (21)

From Equations 17, 18, 20, 21, we have

$$\frac{{\underline{\chi }}_{k+1}\rho_{k+1}+e\left(k+1\right)}{{\bar{\chi }}_{k+1}\rho_{k+1}-e\left(k+1\right)}=\left(1-qT\right)\theta \left(k\right)+qT-\Lambda_{s}sign\left(s\left(k\right)\right)$$ (22)

Then we have (Equation 23) by simplifying (Equation 22).

$$e\left(k+1\right)+a_{1}\Delta y\left(k\right)+\cdots +a_{na}\Delta y\left(k-n_{a}+1\right)$$

$$-b_{1}\Delta u\left(k\right)-\cdots -b_{nb}\Delta u\left(k-n_{b}+1\right)+\tilde{\zeta }\left(k+1\right)$$

$$-\frac{{\bar{\chi }}_{k+1}\left[\left(1-qT\right)\theta \left(k\right)+qT-\Lambda_{s}sign\left(s\left(k\right)\right)\right]-{\underline{\chi }}_{k+1}}{\left(1-qT\right)\theta \left(k\right)+qT+1-\Lambda_{s}sign\left(s\left(k\right)\right)}\rho_{k+1}=0$$ (23)

where $\Delta y\left(k-i\right)=y\left(k-i\right)-y\left(k-1-i\right)$ , (*i* = 0,⋯,*n*_*a*_) and $\Delta u\left(k-j\right)=u\left(k-j\right)-u\left(k-1-j\right)$ , (*j* = 0,⋯,*n*_*b*_). Because the unmodeled dynamics estimation error $\tilde{\zeta }\left(k\right)$ is unknown in experiment (Nguyen et al., 2017), we can obtain the control output (Equation 24) through solving (Equation 23) with the absence of $\tilde{\zeta }\left(k\right)$ .

$$\begin{array}{ll} \Delta u\left(k\right) & =b_{1}^{-1}\left[y_{d}\left(k+1\right)+a_{1}y\left(k\right)+\cdots +a_{na}y\left(k-n_{a}+1\right)-b_{2}u\left(k-1\right)\right.\\ & \;-\cdots -b_{nb}u\left(k-n_{b}+1\right)-\hat{\zeta }\left(k+1\right)\\ & \;\left.-\frac{{\bar{\chi }}_{k+1}\left[\left(1-qT\right)\theta \left(k\right)+qT-\Lambda_{s}sign\left(s\left(k\right)\right)\right]-{\underline{\chi }}_{k+1}}{\left(1-qT\right)\theta \left(k\right)+qT+1-\Lambda_{s}sign\left(s\left(k\right)\right)}\rho_{k+1}\right]\\ & \;=b_{1}^{-1}\left[e\left(k+1\right)+a_{1}\Delta y\left(k\right)+\cdots +a_{na}\Delta y\left(k-n_{a}+1\right)\right.\\ & \;-b_{2}\Delta u\left(k-1\right)-\cdots -b_{nb}\Delta u\left(k-n_{b}+1\right)\\ & \;\left.-\frac{{\bar{\chi }}_{k+1}\left[\left(1-qT\right)\theta \left(k\right)+qT-\Lambda_{s}sign\left(s\left(k\right)\right)\right]-{\underline{\chi }}_{k+1}}{\left(1-qT\right)\theta \left(k\right)+qT+1-\Lambda_{s}sign\left(s\left(k\right)\right)}\rho_{k+1}\right] \end{array}$$ (24)

To further study the principle of controller (Equation 24), we separate the controller into two parts as follows:

$$\begin{array}{ll} ∆u_{linear}\left(k\right) & ={b_{1}}^{-1}\left[e\left(k+1\right)+a_{1}∆y\left(k\right)+\cdots +a_{na}∆y\left(k-n_{a}+1\right)\right.\\ & \;\left.-b_{2}∆u\left(k-1\right)-\cdots -b_{nb}∆u\left(k-n_{b}+1\right)\right] \end{array}$$ (25)

$$∆u_{nonlinear}\left(k\right)=-b_{1}^{-1}\frac{{\underline{\chi }}_{k+1}-{\bar{\chi }}_{k+1}\left[\left(1-qT\right)\theta \left(k\right)+qT-\Lambda_{s}sign\left(s\left(k\right)\right)\right]}{\left(1-qT\right)\theta \left(k\right)+qT+1-\Lambda_{s}sign\left(s\left(k\right)\right)}\rho_{k+1}$$ (26)

where $\Delta u_{\text{linear}}\left(k\right)$ is the linear part which places all the poles of the system to the original point and it can be regarded as one-step-ahead control (OSAC); $\Delta u_{\text{nonlinear}}\left(k\right)$ denotes a nonlinear part which keeps system output not beyond the prescribed domain, although it may cause the output of system away from the desired trajectory. We name (Equation 24) by OSAC-PPC and name (Equation 25) by OSAC for conveniently discussion in the simulations.

Then the control law (Equation 24) can be rewritten as Equation 27

$$u\left(k\right)=u\left(k-1\right)+\Delta u_{\text{linear}}\left(k\right)+\Delta u_{\text{nonlinear}}\left(k\right)$$ (27)

Theorem 1 Consider the nonlinear system (Equation 1) with sliding control law (Equation 24). If the initial parameters ${\bar{\chi }}_{0}$ , ${\underline{\chi }}_{0}$ and $\rho_{0}$ are properly selected to satisfy $-\delta -{\underline{\chi }}_{0}\rho_{0}<e\left(0\right)<{\bar{\chi }}_{0}\rho_{0}+\delta$ , the prescribed performance described by Equation 14 will be guaranteed for all k>0 .

Proof: The proof of Theorem 1 is given in the Appendix.

Remark 1 Similar to the existing PPC approaches in Nguyen et al. (2017) and Liu and Yang (2018), Liu and Yang (2019a), Liu and Yang (2019b), this paper discusses a transformed error algorithm combined with a new sliding mode control strategy to guarantee the tracking error converges to a predefined zone. The control law is separated into two parts: the linear part and nonlinear part. The linear part defined as $\Delta u_{\text{linear}}\left(k\right)$ in this note is to stabilize the system by placing the poles of the system to origin point for the optimal control performance. The nonlinear part defined as $\Delta u_{nonlinear}\left(k\right)$ in this note keeps the output trajectory not crossing the boundaries of the prescribed domain based on the linear part. More precisely, the actual output zone is comparatively enlarged when we add the nonlinear part into the principal linear part of the controller, whereas the output error is still guaranteed within the prescribed zone which is depicted by $\rho_{0}$ , $\rho_{\infty }$ κ , ${\underline{\chi }}_{k}$ and ${\bar{\chi }}_{k}$ . In other words, the linear part is the key to guarantee the tracking error convergent to a predefined arbitrarily small zone with convergence rate no less than a preassigned value and the maximum overshoot less than a desired constant. To this end, we can replace the linear part with any other kind of model-based control methods, such as PID, self-tuning control and Model predictive control (MPC), etc., as long as they are able to guarantee the tracking error within a sufficient small range, so as to accommodate the effects caused by nonlinear part for the achievement of the prescribed performance in Nguyen et al. (2017) and Liu and Yang (2018), Liu and Yang (2019a), Liu and Yang (2019b).

On the other hand, to avoid the estimated parameter ${\hat{b}}_{1}$ close to zero or to change the control effect of the linear part, we can modify the OSAC-PPC (24) into (28) with introducing the parameter *λ* in denominator as follow:

$$∆u\left(k\right)=\frac{\hat{b_{1}}}{\lambda +\hat{{b_{1}}^{2}}}\left[\begin{array}{l} e\left(k+1\right)+\hat{a_{1}}∆y\left(k\right)+\cdots +\hat{a_{na}}∆y\left(k-n_{a}+1\right)\\ -\hat{b_{2}}∆u\left(k-1\right)-\cdots -\hat{b_{nb}}∆u\left(k-n_{b}+1\right)\\ -\frac{{\bar{\chi }}_{k+1}\left[\left(1-qT\right)\theta \left(k\right)+qT-\Lambda_{s}sign\left(s\left(k\right)\right)\right]-{\underline{\chi }}_{k+1}}{\left(1-qT\right)\theta \left(k\right)+qT+1-\Lambda_{s}sign\left(s\left(k\right)\right)}\rho_{k+1} \end{array}\right]$$ (28)

where ${\hat{a}}_{1},\cdots ,{\hat{a}}_{na},{\hat{b}}_{1},\cdots ,{\hat{b}}_{nb}$ represent the estimated values of $a_{1},\cdots ,a_{na},b_{1},\cdots ,b_{nb}$ , respectively. Then the modified controller (Equation 28) has the identical structure with those in Liu and Yang (2018), Liu and Yang (2019a), Liu and Yang (2019b). And we name (Equation 28) by improved OSAC-PPC.

## 4 Experiment and result

**FIGURE 1:**
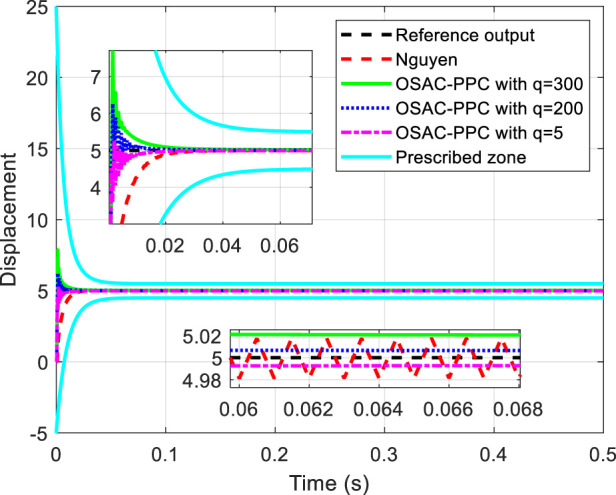
Tracking performance comparisons and convergence zone.

**FIGURE 2:**
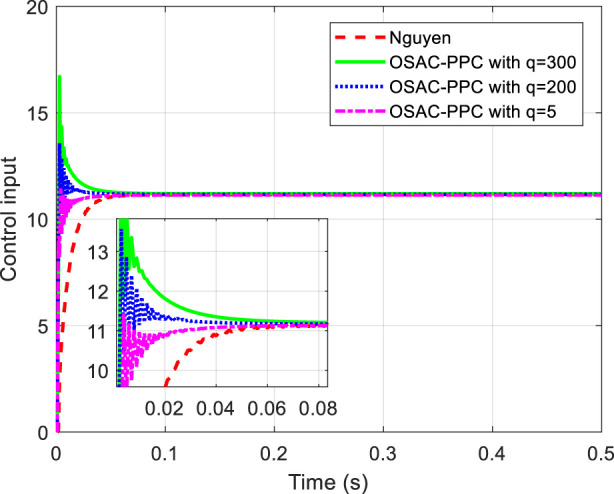
Control input of each controller.

Example 1 Consider the system (Equation 29) in Nguyen et al. (2017).

$$y\left(k+1\right)=a_{1}y\left(k\right)-a_{2}y\left(k-1\right)+b_{1}u\left(k\right)+b_{2}u\left(k-1\right)+\zeta \left(k+1\right)$$ (29)

where *a*_1_ = −0.1903, *a*_2_ = −0.00906, *b*_1_ = 0.4906 and *b*_2_ = 0.04723. We assume *ζ*(*k*) = 0 at beginning to exhibit the effects of controllers more clearly. All the simulation settings including reference output, convergence zone described by Equation 14, and the controller parameters *T* = 0.0005, κ=0.05 , $\rho_{0}=10$ , $\rho_{\infty }=0.5$ , ${\underline{\chi }}_{0}=1$ and ${\bar{\chi }}_{0}=2$ are chosen in common with Nguyen et al. (2017). The proposed OSAC-PPC method (24) choose controller parameter *q* with different values: 300, 200 and 5. To be consistent with Nguyen et al. (2017). Figure 1 shows the step response comparison between the proposed OSAC-PPC method and the Nguyen’s method. Control input of each controller is shown in Figure 2.

Figure 1 obviously shows that each output of system controlled by these methods is in prescribed zone. From Figure 1, we can see that: i) from the time of [0, 0.02], the tracking error of system controlled by the proposed OSAC-PPC method is smaller than that of Nguyen. Besides, we can change the transient performance by adjusting *q*. When the parameter *q* decreases, the convergence speed and the oscillation of system controlled by proposed method will increase; ii) from the time of [0.06, 0.066], the output of system controlled by the proposed OSAC-PPC method with *q* = 5 consistently converges to the desired trajectory; The output of the system controlled by the proposed OSAC-PPC method with *q* = 200 passes through the output trajectory of Nguyen’s controller; The output of the system controlled by the proposed OSAC-PPC method with *q* = 300 is designed so as to be tangent to the actual output zone of Nguyen.

From Figure 1, we can conclude that the tracking error of the proposed method is smaller than that of Nguyen under the same prescribed performance. In addition, by decreasing the controller parameter *q*, we can change the system transient characteristics to obtain a better convergence speed, nevertheless the oscillation is enlarged. Therefore, the proposed OSAC-PPC method is more flexible than Nguyen’s controller for introducing the key adjustable parameter *q*. As a result, we have more choices for the system behaviors and transient characteristics.

**FIGURE 3:**
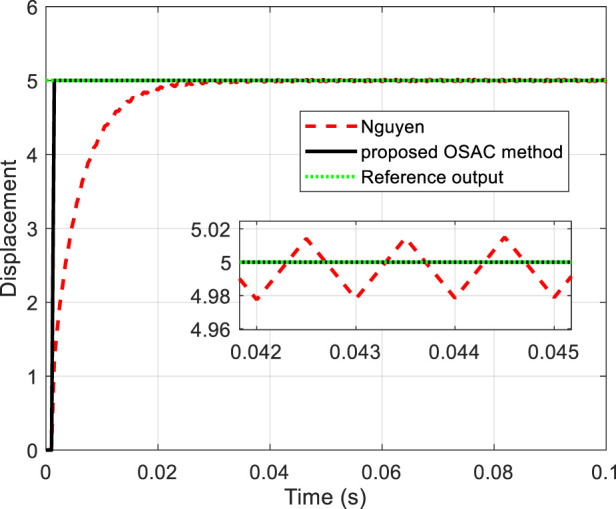
Tracking performance comparison.

On the other hand, to further study this kind of prescribed performance method, we choose $\rho_{0}=0$ and $\rho_{\infty }=0$ with an aim to remove the nonlinear part of the controller, i.e., $\Delta u_{\text{nonlinear}}\left(k\right)=0$ . The output of the system controlled by the only linear part of OSAC-PPC method (Equation 24) [i.e., OSAC (Equation 25)] and Nguyen’s method are shown in Figure 3.

From Figure 3, we can see that the system controlled by OSAC (Equation 25) exhibits a better performance even without overshoot in simulation. It is natural for us to conclude that the effectiveness of the proposed method can be separated into two parts: i) the stability of the system is guaranteed by the linear part of the OSAC-PPC controller (Equation 24) whose intrinsic design is for the achievement of the optimal performance. ii) The actual output zone is virtually widened by the nonlinear part of the controller (Equation 26); however, the system is still guaranteed within the prescribed zone depicted by $\rho_{0}$ , $\rho_{\infty }$ and κ .

**FIGURE 4:**
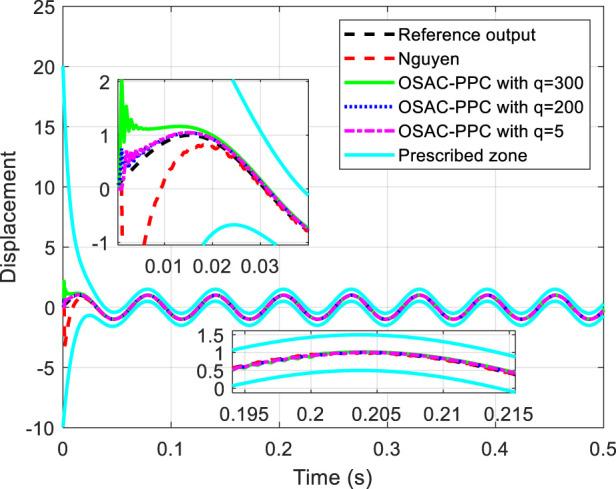
Tracking performance comparisons and convergence zone.

**FIGURE 5:**
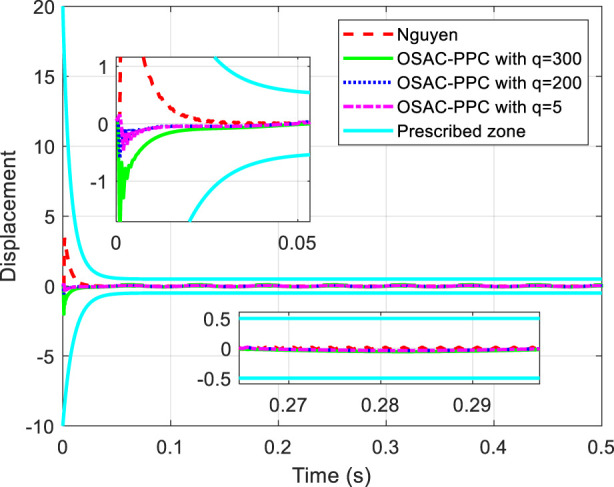
Tracking error and prescribed zone for the error.

**FIGURE 6:**
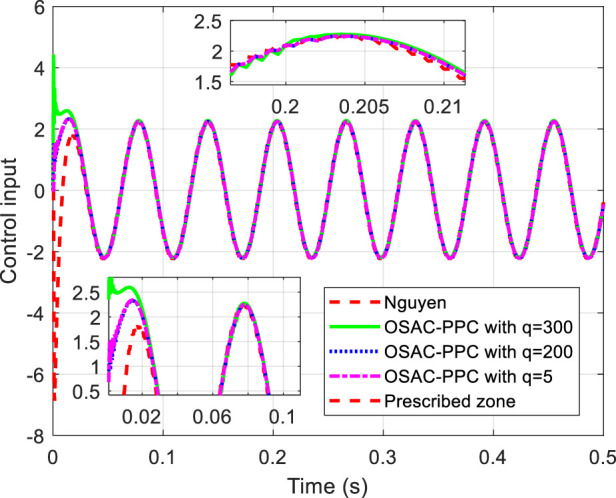
The control input of each controller.

When we change the reference trajectory to $y\left(k\right)=\sin\left(2\times 10^{‐5}t\right)$ , while keeping the controller parameters unchanged, the corresponding tracking performance is depicted in Figure 4. The tracking error is shown in Figure 5, and the control inputs are illustrated in Figure 6.

From Figures 4, 5, it can be observed that both the proposed OSAC-PPC method and Nguyen’s method keep the system outputs and tracking errors within the prescribed zone. Moreover, the proposed OSAC-PPC method exhibits a smaller tracking error compared to Nguyen’s method.

Example 2 This example shows that the improved OSAC-PPC (Equation 28) are more suitable for the case of inaccuracy of estimate parameters. We assume that the inaccurately offline estimated parameters are ${\hat{a}}_{1}=a_{1}+0.3$ , ${\hat{a}}_{2}=a_{2}+0.7$ , ${\hat{b}}_{1}=b_{1}-0.1$ and ${\hat{b}}_{2}=b_{2}-0.02$ in Example 1. Then the corresponding unmodeled dynamics will be Equation 30

$$\zeta \left(k+1\right)=-0.3y\left(k\right)-0.7y\left(k-1\right)+0.1u\left(k\right)+0.02u\left(k-1\right)$$ (30)

**FIGURE 7:**
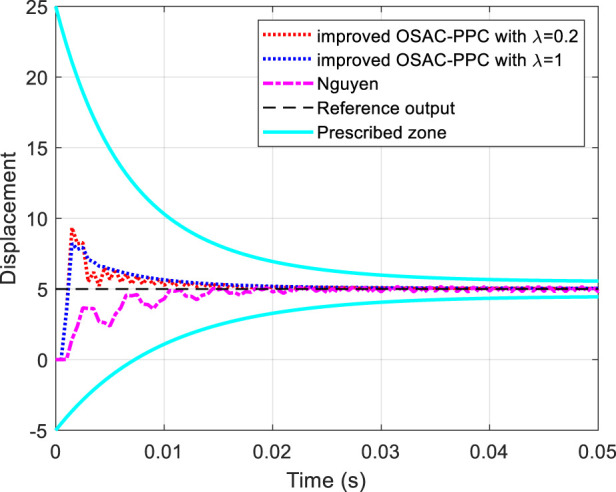
Tracking performance comparisons and convergence.

**FIGURE 8:**
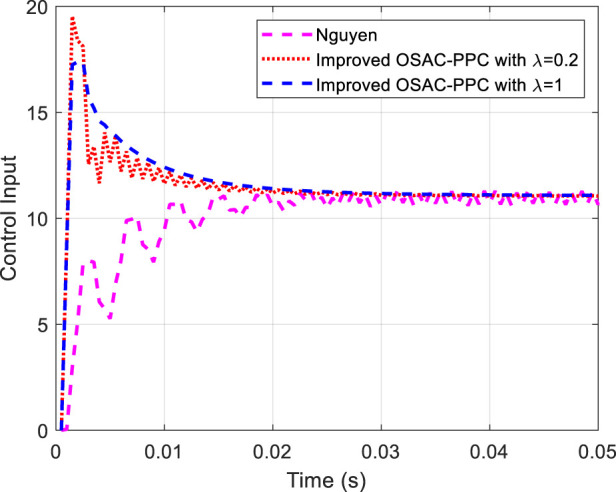
Control input of each controller.

The output of the system controlled by Nguyen’s controller and improved OSAC-PPC (28) with *λ* = 0.2 and *λ* = 1 is shown in Figure 7, respectively. The control input of each controller is shown in Figure 8.

From Figure 7, we can find that the output of the system controlled by Nguyen’s controller or improved OSAC-PPC (28) with *λ* = 0.2 oscillates at the beginning owing to the estimate parameter error. By comparison, when we choose *λ* = 1, the output oscillation of the system controlled by improved OSAC-PPC (Equation 28) is eliminated. On the other hand, Figure 8 also shows that the control input of Nguyen oscillates obviously from beginning to end. By comparison, the oscillation of the control input of improved OSAC-PPC (28) is eliminated through letting *λ* = 1, since the increment of penalty constant *λ* constrains the change of the control output. Owing to the system’s transient characteristics can be changed through adjusting the introduced penalty constant *λ*, the improved OSAC-PPC (28) are more stable and suitable for the cases of inaccuracy of estimate parameters.

There are many well-known performance indexes for the system, we should not focus on only one index (i.e., prescribed performance) but should consider more system transient indexes such as system convergent speed, smoothness, overshoot and so on. These examples show that some transient performances can be improved by our improvements meanwhile the prescribed performance is still satisfied.

Owing to the system’s transient characteristics can be changed through adjusting the introduced penalty constant *λ*, Furthermore, we can replace the linear part of improved OSAC-PPC (28) with MPC or self-tuning PID. It will also achieve the comparable performance in the same way.

**FIGURE 9:**
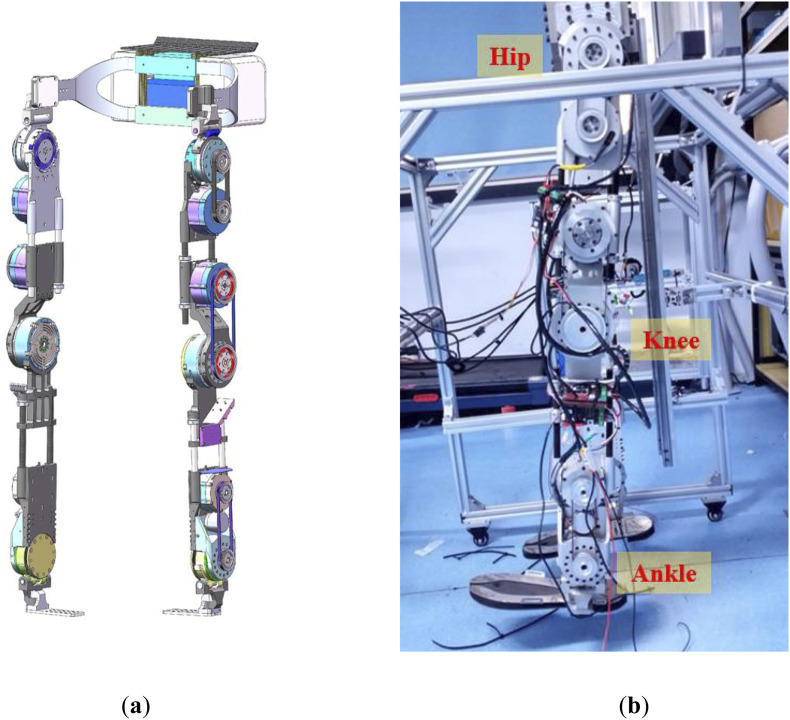
**(A)**Three-dimensional modeling of the lower limb exoskeleton robotic system; **(B)**Three joints lower limb exoskeleton system.

Example 3 We validate the PPC controller in a three joints SEA-based lower limb exoskeleton system, which is shown in Figure 9. The joints of this system are actively controlled through current loops, with each joint’s torque values calculated by the controller and then converted into control inputs. This motor-driven approach enables the exoskeleton to follow the reference trajectories, which are scaled versions of gait cycle joint angles, specifically chosen to test the controller’s performance. The exoskeleton operates on an EtherCAT-based motor drive system.

The platform is equipped with two 24 V serial batteries providing the necessary power for the lower limb exoskeleton. The drive module comprises six modular joints, divided equally between the left and right limbs, corresponding to the human body’s hip, knee, and ankle joints. Absolute encoders installed at each joint measure the angles of rotation.

**FIGURE 10:**
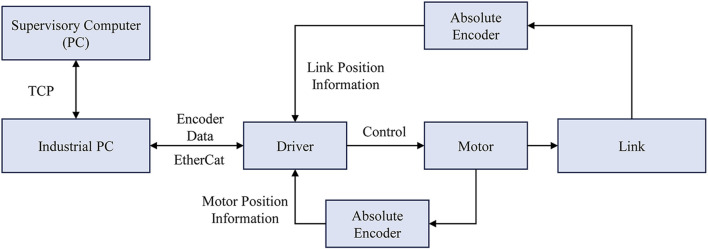
Exoskeleton hardware control architecture.

Figure 10 illustrates the hardware control architecture of the exoskeleton. The heart of the exoskeleton’s control system is an industrial PC (IPC) equipped with an i7 7600U processor running the Ubuntu operating system. This IPC serves as the control center where the core control strategies are implemented. Communication between the IPC and the supervisory computer is established via SSH, with the startup program initiated and control commands sent using TCP communication. The exoskeleton’s status information is transmitted using LCM.

In terms of exoskeleton control commands, the IPC is connected to the drivers and operates as the EtherCAT master using the IgH EtherCAT Master for Linux, while the drivers function as slaves. The communication between the IPC and the exoskeleton includes sending and receiving control commands and sensor information with a control cycle of 1,000 Hz.

To establish a baseline for comparison, we included two control groups in our study. The first control group employs an incremental PID + dynamics controller that we developed, which integrates dynamic feedforward compensation to enhance control performance. The second group uses a traditional incremental PID controller, serving as a baseline to assess the advancements offered by our proposed solutions.

The dynamic model (Equations 31, 32) is given by

$$\left(H\left(q\right)+S^{T}\right)\ddot{q}+\left(B+S\right){\ddot{\theta }}_{M}+C\left(q,\dot{q}\right)\dot{q}+G\left(q\right)+\tau_{f}=u$$ (31)

$$H\left(q\right)=M_{L}\left(q\right)+M_{R}\left(q\right)+S\left(q\right)B^{-1}S^{T}\left(q\right)$$ (32)

where, ***H*** (***q***) is the total inertia matrix; ***B*** is the inertia matrix of the motor considering the reduction ratio; and ***S*** is the motor-link coupling matrix, which is a constant matrix. ***M***_*R*_(***q***) is the added inertia matrix of the connecting link introduced by the motor (Li et al., 2017; Chen et al., 2019; Siciliano and Khatib, 2016).

The dynamic parameters of links are inaccurately identified and given in Table 1.

**TABLE 1:** Dynamic parameters of exoskeleton.

Parameters	Hip (1)	Knee (2)	Angle (3)
*m*_*i*_(*kg*)	2.09	1.6	0.55
*m*_*i*_ *L*_*ci*_(*kg•m*)	0.1567	0.0689	0.0024
*L*_*i*_(*m*)	0.37	0.36	∗
*J*_*i*_(*kg•m* ^2^)	−0.304	0.0002	0.0097
*f*_*ci*_(*Nm*)	0.0847	0.6758	1.7567
*f*_*vi*_(*Nm•s/rad*)	−3.2967	−3.7335	−12.2408

Since the leading coefficient of control input is an identity matrix, we can describe the linear part of the controller as

$$\begin{array}{ll} \Delta u_{\text{linear}}\left(k\right) & =K_{P}\left[e\left(k+1\right)-e\left(k\right)\right]+K_{I}e\left(k+1\right)+\left(H\left(q\left(k\right)\right)+S^{T}\right)\ddot{q}\left(k\right)\\ & \;+\left(B+S\right){\ddot{\theta }}_{M}\left(k\right)+C\left(q\left(k\right),\dot{q}\left(k\right)\right)\dot{q}\left(k\right)+G\left(q\left(k\right)\right)+\tau_{f}\\ & \;-\left[\left(H\left(q\left(k-1\right)\right)+S^{T}\right)\ddot{q}\left(k-1\right)+\left(B+S\right){\ddot{\theta }}_{M}\left(k-1\right)\right.\\ & \;\left.+C\left(q\left(k-1\right),\dot{q}\left(k-1\right)\right)\dot{q}\left(k-1\right)+G\left(q\left(k-1\right)\right)+\tau_{f}\right] \end{array}$$ (33)

where, $K_{P}=diag\left(k_{P}^{1},k_{P}^{2},k_{P}^{3}\right)$ and $K_{I}=diag\left(k_{I}^{1},k_{I}^{2},k_{I}^{3}\right)$ .

Meanwhile, the nonlinear part can be described as

$$\Delta u_{\text{nonlinear}}\left(k\right)=\left[\begin{array}{c} \frac{{\bar{\chi }}_{k+1}^{1}\left[\left(1-q^{1}T\right)\theta^{1}\left(k\right)+q^{1}T-\Lambda_{s}^{1}sign\left(s^{1}\left(k\right)\right)\right]-{\underline{\chi }}_{k+1}^{1}}{\left(1-q^{1}T\right)\theta^{1}\left(k\right)+q^{1}T+1-\Lambda_{s}^{1}sign\left(s^{1}\left(k\right)\right)}\rho_{k+1}^{1}\\ \frac{{\bar{\chi }}_{k+1}^{2}\left[\left(1-q^{2}T\right)\theta^{2}\left(k\right)+q^{2}T-\Lambda_{s}^{2}sign\left(s^{2}\left(k\right)\right)\right]-{\underline{\chi }}_{k+1}^{2}}{\left(1-q^{2}T\right)\theta^{2}\left(k\right)+q^{2}T+1-\Lambda_{s}^{2}sign\left(s^{2}\left(k\right)\right)}\rho_{k+1}^{2}\\ \frac{{\bar{\chi }}_{k+1}^{3}\left[\left(1-q^{3}T\right)\theta^{3}\left(k\right)+q^{3}T-\Lambda_{s}^{3}sign\left(s^{3}\left(k\right)\right)\right]-{\underline{\chi }}_{k+1}^{3}}{\left(1-q^{3}T\right)\theta^{3}\left(k\right)+q^{3}T+1-\Lambda_{s}^{3}sign\left(s^{3}\left(k\right)\right)}\rho_{k+1}^{3} \end{array}\right]$$ (34)

where, ${*}^{i}$ represent the parameters for *i*-th joint.

Then we propose an incremental PID + dynamics based PPC controller as Equation 35

$$u\left(k\right)=u\left(k-1\right)+\Delta u_{\text{linear}}\left(k\right)+\Delta u_{\text{nonlinear}}\left(k\right)$$ (35)

The controllers used for comparison are incremental PID + dynamics feedforward control (Equation 33), and incremental PID (Equation 36).

$$\Delta u_{\text{PID}}\left(k\right)=K_{P}\left[e\left(k+1\right)-e\left(k\right)\right]+K_{I}e\left(k+1\right)$$ (36)

It should be noted that the incremental PID + dynamics-based PPC controller is obtained by summing the PID + dynamics feedforward compensation controller (Equation 33) with the nonlinear part (Equation 34). Both controllers use dynamics feedforward compensation in differential form.

**FIGURE 11:**
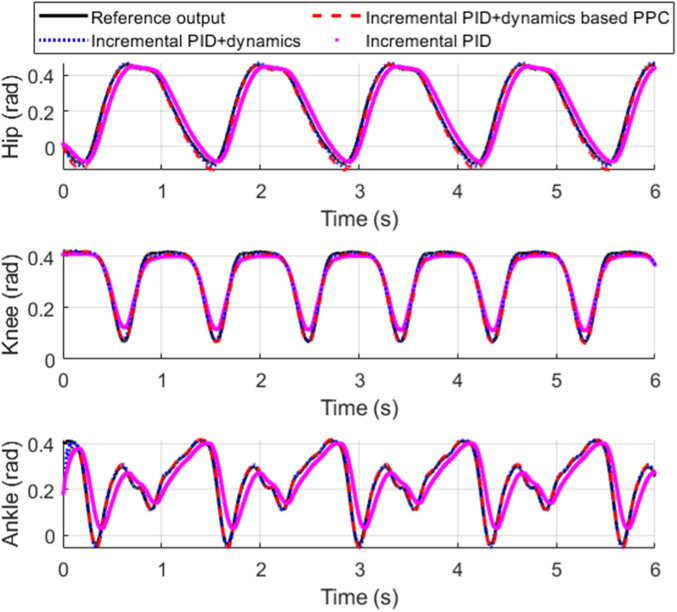
Tracking performance comparisons.

**FIGURE 12:**
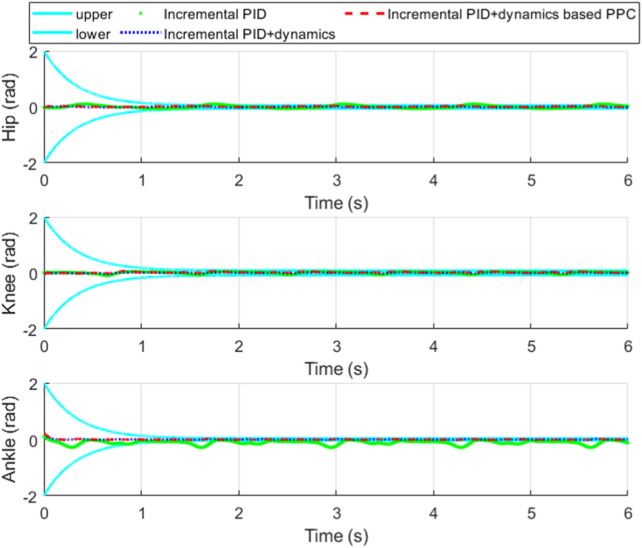
Tracking error and its convergence zone.

**FIGURE 13:**
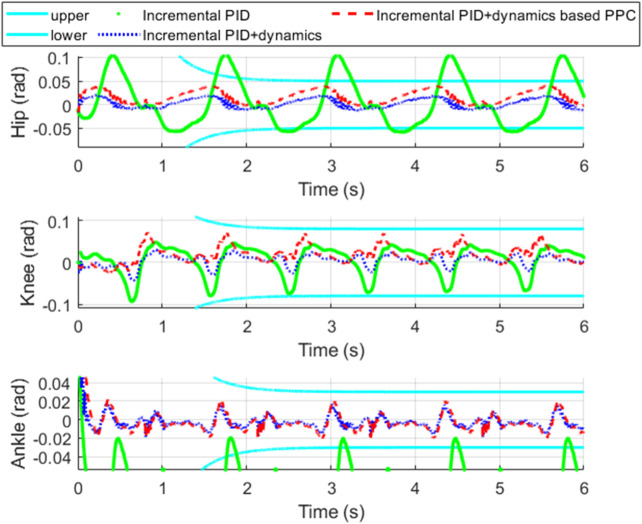
Enlarged view of Figure 12.

**FIGURE 14:**
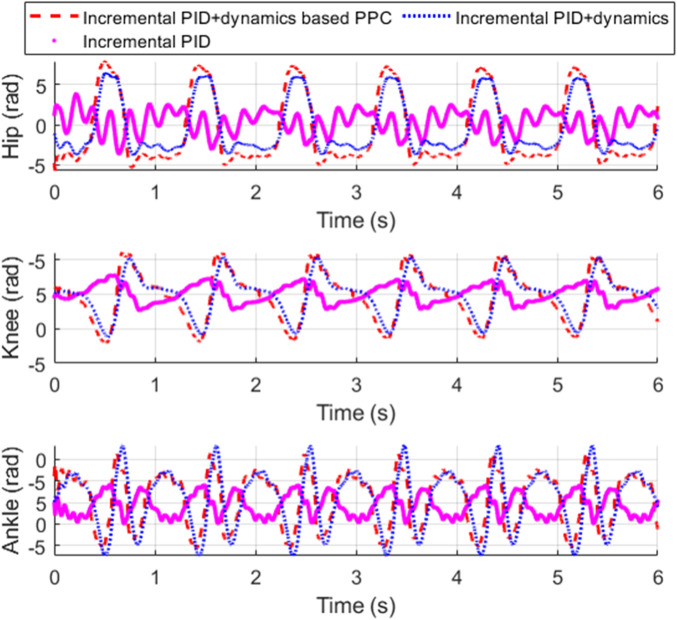
Control input of each controller.

The controller parameters are given by Table 2. The sampling time *T* = 0.001*s.* Figure 11 shows the tracking performance of each joint of the exoskeleton. Figure 12 shows the tracking error of each joint and Figure 13 shows the enlarged view of Figure 12. The control input of each joint is shown in Figure 14.

**TABLE 2:** Controller parameters.

Parameters	Joint 1	Joint 2	Joint 3
*q* ^*i*^	5	5	5
κ	0.001	0.001	0.001
${\bar{\chi }}_{0}^{i}$	1	1	1
${\underline{\chi }}_{0}^{i}$	1	1	1
$\rho_{0}^{i}$	2	2	2
$\rho_{\infty }^{i}$	0.05	0.05	0.03
$k_{P}^{i}$	300	300	400
$k_{I}^{i}$	3	1.5	4

From Figure 11, it is evident that compared to incremental PID, both incremental PID + dynamics-based PPC controller and incremental PID + dynamics controller significantly enhance the tracking accuracy of the three joints. This indicates that our designed differential form of dynamics-based feedforward effectively compensates for the accuracy deficiencies caused by the low stiffness and elastic characteristics of the SEA. From Figures 12, 13, we can observe that the system controlled by the incremental PID + dynamics-based PPC controller does not perform superior to the incremental PID + dynamics controller. However, the tracking errors for both remain within the prescribed zone. The design of the PPC is not intended to enhance the controller’s performance; rather, control accuracy and response time might be reduced due to the prescribed performance index or constraint of the tracking error. Despite this, the method ensures that errors are maintained within the prescribed zone limits.

## 5 Conclusion

In this paper, we propose a discrete-time sliding-mode control method based on prescribed performance control method to cope with a kind of tracking error constrained problem in the discrete-time dynamical system. By separating controller into two parts, we analyzed the principle of a family of PPC methods which aim to ensure that the tracking error converges to a predefined region. Moreover, we give some alternative improvements for the linear part of the controller for its alterable performance and yet not to impact the premise of prescribed performance. Simulations and experiments are provided to validate the established results and the effectiveness of the proposed method.

## Data Availability

The datasets presented in this article are not readily available because The data that support the findings of this study are available on request from the author, upon reasonable request. Requests to access the datasets should be directed to Feilong Zhang, zhangfeilong@sia.cn.

## Proof: Appendix of Theorem 1

If the tracking error $e\left(k\right)$ satisfies $-\delta -{\underline{\chi }}_{k}\rho_{k}<e\left(k\right)<{\bar{\chi }}_{k}\rho_{k}+\delta$ at any time *k*, through the proposed controller (Equation 24), we want to demonstrate that one step ahead tracking error $e\left(k+1\right)$ also satisfies the prescribed performance control: $-\delta -{\underline{\chi }}_{k+1}\rho_{k+1}<e\left(k+1\right)<{\bar{\chi }}_{k+1}\rho_{k+1}+\delta$ . Thereafter, the control algorithm can be repeated in the next sampling cycle.

To demonstrate this result, based on Equations 18–21, 23, we have

$$e\left(k+1\right)=\frac{{\bar{\chi }}_{k+1}\left[\left(1-qT\right)\theta \left(k\right)+qT-\Lambda_{s}sign\left(s\left(k\right)\right)\right]-{\underline{\chi }}_{k+1}}{\left(1-qT\right)\theta \left(k\right)+qT+1-\Lambda_{s}sign\left(s\left(k\right)\right)}\rho_{k+1}+\xi \left(k+1\right)$$
(A1)

From Equations 21, A1, we can obtain Equation A2.

$$e\left(k+1\right)<\frac{{\bar{\chi }}_{k+1}\left[\left(1-qT\right)\theta \left(k\right)+qT+\Lambda_{s}\right]-{\underline{\chi }}_{k+1}}{\left(1-qT\right)\theta \left(k\right)+qT+1+\Lambda_{s}}\rho_{k+1}+\delta$$
(A2)

Obviously, $\frac{{\bar{\chi }}_{k+1}\left[\left(1-qT\right)\theta \left(k\right)+qT+\Lambda_{s}\right]-{\underline{\chi }}_{k+1}}{\left(1-qT\right)\theta \left(k\right)+qT+1+\Lambda_{s}}\rho_{k+1}$ is a function with respect to $\theta \left(k\right)$ and is monotonic increasing with $\theta \left(k\right)$ . Therefore, we have

$$e\left(k+1\right)<\lim_{\theta \left(k\right)\to \infty }\frac{{\bar{\chi }}_{k+1}\left[\left(1-qT\right)\theta \left(k\right)+qT-\Lambda_{s}\right]-{\underline{\chi }}_{k+1}}{\left(1-qT\right)\theta \left(k\right)+qT+1+\Lambda_{s}}\rho_{k+1}+\delta$$

$$={\bar{\chi }}_{k+1}\rho_{k+1}+\delta$$
(A3)

With the help of Equations 21, A1, we have Equation A4.

$$e\left(k+1\right)>\frac{{\bar{\chi }}_{k+1}\left[\left(1-qT\right)\theta \left(k\right)+qT-\Lambda_{s}\right]-{\underline{\chi }}_{k+1}}{\left(1-qT\right)\theta \left(k\right)+qT+1-\Lambda_{s}}\rho_{k+1}-\delta$$
(A4)

Similar to the proof process of (Equation A3), we can infer that

$$e\left(k+1\right)>\lim_{\theta \left(k\right)\to 0}\frac{{\bar{\chi }}_{k+1}\left[qT-\Lambda_{s}\right]-{\underline{\chi }}_{k+1}}{qT+1-\Lambda_{s}}\rho_{k+1}-\delta =-{\underline{\chi }}_{k+1}\rho_{k+1}-\delta$$
(A5)

From (Equations A3, A5), it can be concluded that

$$-\delta -{\underline{\chi }}_{k+1}\rho_{k+1}<e\left(k+1\right)<{\bar{\chi }}_{k+1}\rho_{k+1}+\delta$$
(A6)
